# Derivation of Corrosion Depth Formula According to Corrosion Factors in District Heating Water through Regression Analysis

**DOI:** 10.3390/ma16083254

**Published:** 2023-04-20

**Authors:** Yoon-Sik So, Jeong-Min Lim, Sin-Jae Kang, Woo-Cheol Kim, Jung-Gu Kim

**Affiliations:** 1 School of Advanced Materials Science and Engineering, Sungkyunkwan University, Suwon 16419, Republic of Korea; soy4718@skku.edu (Y.-S.S.); alsdl0311@skku.edu (J.-M.L.); paul8121@naver.com (S.-J.K.); 2 Plant Management and QC Division, Korea District Heating Corporation, Sungnam 13585, Republic of Korea; kwc7777@kdhc.co.kr

**Keywords:** galvanostatic test method, underground infrastructure, long-term corrosion, carbon steel, weldment

## Abstract

In order to predict the corrosion depth of a district heating pipeline, it is necessary to analyze various corrosion factors. In this study, the relationship between corrosion factors such as pH, dissolved oxygen, and operating time and corrosion depth was investigated using the Box–Behnken method within the response surface methodology. To accelerate the corrosion process, galvanostatic tests were conducted in synthetic district heating water. Subsequently, a multiple regression analysis was performed using the measured corrosion depth to derive a formula for predicting the corrosion depth as a function of the corrosion factors. As a result, the following regression formula was derived for predicting the corrosion depth: “corrosion depth (μm) = −133 + 17.1 pH + 0.00072 DO + 125.2 Time − 7.95 pH × Time + 0.002921 DO × Time”.

## 1. Introduction

District heating (DH) pipelines are designed for long-term use because they are difficult to maintain or replace [1,2]. Welding is commonly used for constructing long-distance DH pipelines. However, when carbon steel pipes are welded, the microstructure of the weldment differs from that of the base metal, leading to dissimilarity in individual parts of the weldment. This dissimilarity induces corrosion between the heat-affected zone (HAZ) and the weld metal when they are exposed to corrosive environments. DH pipelines are used for supplying heat using hot DH water, which exposes them to corrosive environments. Therefore, it is crucial to determine the corrosion behavior of buried pipelines [3,4,5]. Various factors, such as pH and dissolved oxygen [6,7,8], threaten the stable maintenance of DH pipelines. To prevent failure and predict service life, it is necessary to examine the corrosion characteristics of DH pipelines in real environments [9]. However, there are various types of corrosion factors present in DH water [10], and the concentration of each factor is constantly changing, making it challenging to examine the corrosion rate under all conditions. As a result, several studies have been conducted to predict the effect of corrosion under various types and concentrations using a statistical analysis approach [11,12]. To use this statistical analysis, the design of experiment (DOE) is necessary. Among the DOE methods, response surface methodology (RSM) is beneficial for efficiently evaluating the effects of various factors and reducing the number of experiments [13,14,15]. Therefore, this research aims to examine the corrosion factors (pH and DO) present in DH water and the corrosion depth of the DH pipeline welded joint based on the operating time using RSM.

## 2. Materials and Methods

### 2.1. Specimen and Solution

**Figure 1:**
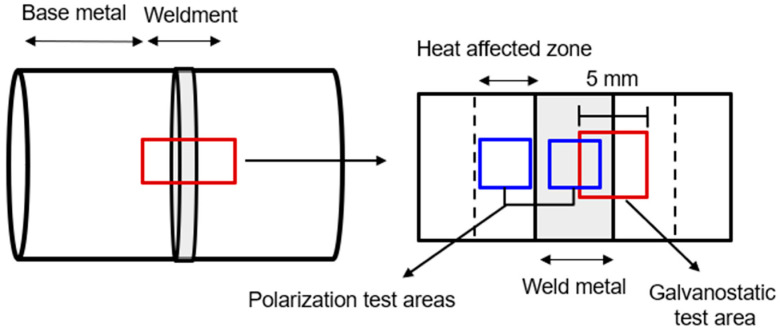
Schematic illustration of the sectioning procedures used to prepare the specimen from the DH pipeline weldment.

A test specimen consisting of the weldment of the DH pipeline was utilized, as depicted in Figure 1. This specimen comprises of a heat-affected zone and weld metal [10]. Prior to testing, the specimen was polished with 600-grit SiC paper and dried with N_2_. Next, the specimen was sealed with silicon, exposing an area of 5 mm × 5 mm, which included both the heat-affected zone and weld metal. The chemical composition of the DH pipeline steel (SPW400) is presented in Table 1. The test solution used was synthetic DH water, and its composition is shown in Table 2.

### 2.2. Electrochemical Tests

All electrochemical tests were performed using a multipotentiostat/galvanostat instrument (VMP-2, Bio-Logic Science Instruments, Seyssinet-Pariset, France) with a three-electrode system. The tested specimen served as the working electrode (WE), while two pure graphite rods served as the counter electrode (CE, Qrins, Seoul, Korea), and a saturated calomel electrode was used as the reference electrode (RE, Qrins, Seoul, Korea). A DO sensor was utilized to maintain a suitable DO level during the experiment with a 3 W motor circulating the solution. The level of DO was adjusted by purging a mixed gas (99.9% N_2_ and 0.1% O_2_). Prior to the electrochemical tests, the specimens were immersed in a test solution for 3 h to obtain a stable open-circuit potential, and the DO levels were monitored using a display unit. Potentiodynamic polarization measurements were conducted according to ASTM G5, with a potential sweep rate of 0.166 mV/s from the open-circuit potential (OCP) to −0.25 V vs. OCP and from the OCP to +0.40 V vs. OCP. To accelerate corrosion, a galvanostatic test [16] was performed on a set of 60 tests. In the galvanostatic test, an impressed anodic current density of 4 mA/cm^2^ was applied during the testing time, which is calculated by the corrosion current density of HAZ using Faraday’s law.

### 2.3. Surface Analysis

**Figure 2:**
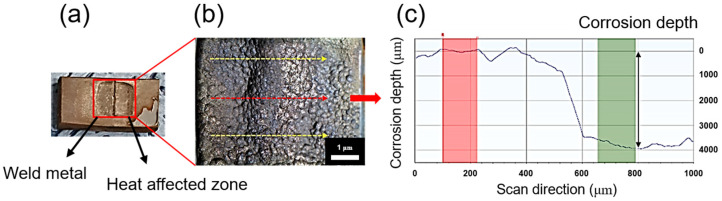
Corrosion depth examination process using a 3D profiler. (**a**) The corroded weldment after the galvanostatic test, (**b**) magnified image of the red square in (**a**), and (**c**) corrosion depth measurement.

The microstructure of the weldment was examined using an optical microscope (SZ61TRC, Olympus Korea Co., Seocho-gu, Seoul, Korea). After the galvanostatic test, an OM was used to observe the surface morphology of each specimen, and the corrosion depth was measured using a surface profiler (DektakXT, BRUKER, North Billerica, MA, USA). The procedure for measuring the corrosion depth is illustrated in Figure 2. Prior to measuring the corrosion depth, the silicon and corrosion products were removed from the specimen by mechanical cutting and acid cleaning. The corrosion depth was measured 3–5 times, depending on the surface condition, and the average corrosion depth was used as data for the regression analysis.

### 2.4. Regression Analysis

RSM is a statistical and mathematical tool that uses DOE to analyze the effect of variables [17]. The response surface is a function that relates the response to independent variables. This relationship is derived through regression analysis based on the DOE. In this study, 60 experiments were performed using pH (7, 8, 9, 10, and 11), DO (0, 200, 1000, and 8000 ppb), and operating time (2.5, 12.5, and 25 years) as independent variables. The experimental procedure was designed using the Box–Behnken design. The response surface equation was estimated using multiple regression analysis based on the measured data. A model including Equation (1) was used for the regression analysis [18].

Y = *f* (X_1_, X_2_, X_3_) ± E (1)

where Y represents the estimated corrosion depth, *f* is a response function, X_1_, X_2_, and X_3_ are the experimental factors (pH, DO, and time-of-use, respectively), and E represents an experimental error [19]. This model is a general polynomial with an unknown structure; however, it is suitable to use a second-order polynomial model [20]. The estimated corrosion depth was related to the variables by the second-order polynomial regression model given in Equation (2):

(2) $$Y=\beta_{0}+\sum_{i=1}^{n}\beta_{i}X_{i}+\sum_{i=1}^{n}\beta_{ii}X_{i}^{2}+\sum_{i=1}^{n-1}\sum_{j>i}^{n}\beta_{ij}X_{i}X_{j}+E$$

where β_0_ represents an intercept or regression coefficient; β*_i_*, β*_ii_*, and β*_ij_* represent the linear, quadratic, and intercept parameters, respectively; *X_i_* and *X_j_* are the process variables’ coded values [19]. The fit quality of this model was evaluated by the coefficient of determination (R^2^) and analysis of variance (ANOVA).

### 2.5. District Heating Water Monitoring

The level of pH and dissolved oxygen in the DH water were monitored using a bypass unit connected to the operating pipeline. The bypass unit consists of a flow chamber, a dissolved oxygen sensor (DO probe, Mettler Toledo, Columbus, OH, USA), and a pH meter (3-2716, GF Signet, Irwindale, CA, USA). The data were recorded every 30 min for 250 days.

## 3. Results and Discussion

### 3.1. Microstructure of Weldment

**Figure 3:**
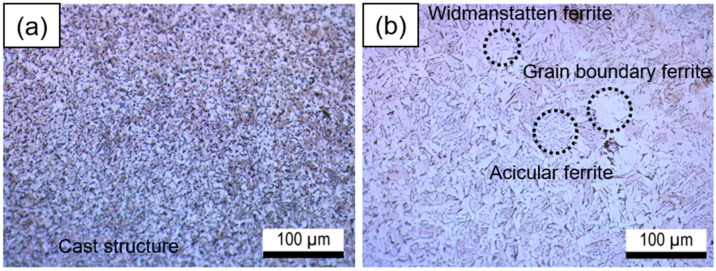
Optical micrographs of the weldment: (**a**) weld metal; (**b**) heat-affected zone.

Figure 3 depicts the optical micrographs of the HAZ and the weld metal. The weld metal exhibited a fine grain size (Figure 3a). However, due to the heat generated during the welding process, the HAZ exhibited a larger grain size than the weld metal (Figure 3b) [21]. The weld metal had a cast structure, while the HAZ was composed of widmanstatten ferrite, grain boundary ferrite, and acicular ferrite. The electrochemical behavior of the HAZ is influenced by the complex microstructures, which may decrease the polarization resistance [7,22,23,24].

### 3.2. Potentiodynamic Test

**Figure 4:**
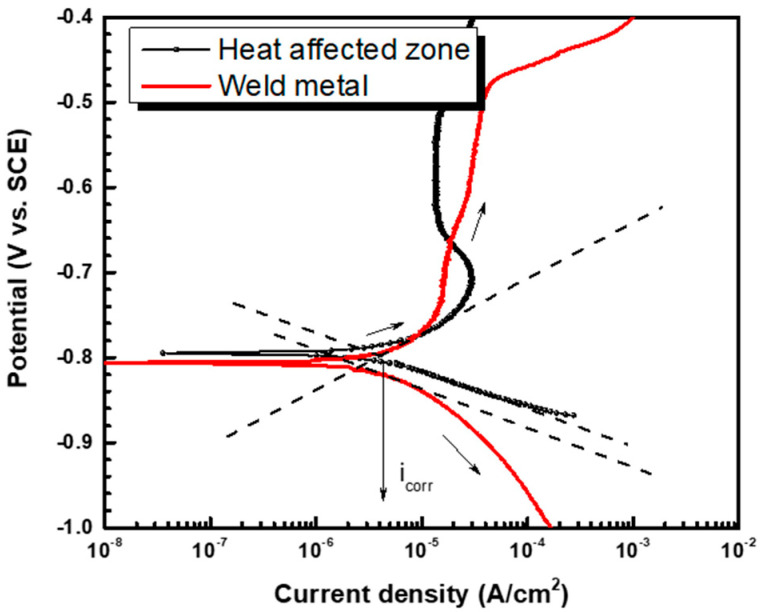
Polarization curves of the heat-affected zone and the weld metal of pipeline steel in the synthetic DH water.

The potentiodynamic test was performed to examine the corrosion current density of the weldment of pipeline steel in DH water, and the results are shown in Figure 4. To compute the corrosion current density, the Tafel extrapolation method was used [25]. Equation (3) describes the linear relationship between the overpotential and the log scale current density:

η = a ± β_(a,c)_log|i|, a = −β_a_log(i_0_) or β_c_log(i_0_), β_a_ ≅ (RT/(1 − α)nF), β_c_ ≅ (RT/αnF) (3)

where β_a_ represents the anodic Tafel slope, β_c_ represents the cathodic Tafel slope, i_0_ represents the exchange current density, α represents the charge transfer coefficient, n represents the charge number, R represents the gas constant (8.314 J/(mol∙K)), and T represents the absolute temperature (K). From Equation (3), a linear relationship was derived, and the corrosion current density of the HAZ was measured at 4.304 μA/cm^2^, and the corrosion current density of the weld metal was measured at 2.569 μA/cm^2^ according to the polarization curves as shown in Figure 4 and Table 3. As a result, the HAZ is more prone to corrosion than the weld metal [26,27].

### 3.3. Corrosion Acceleration Test

**Figure 5:**
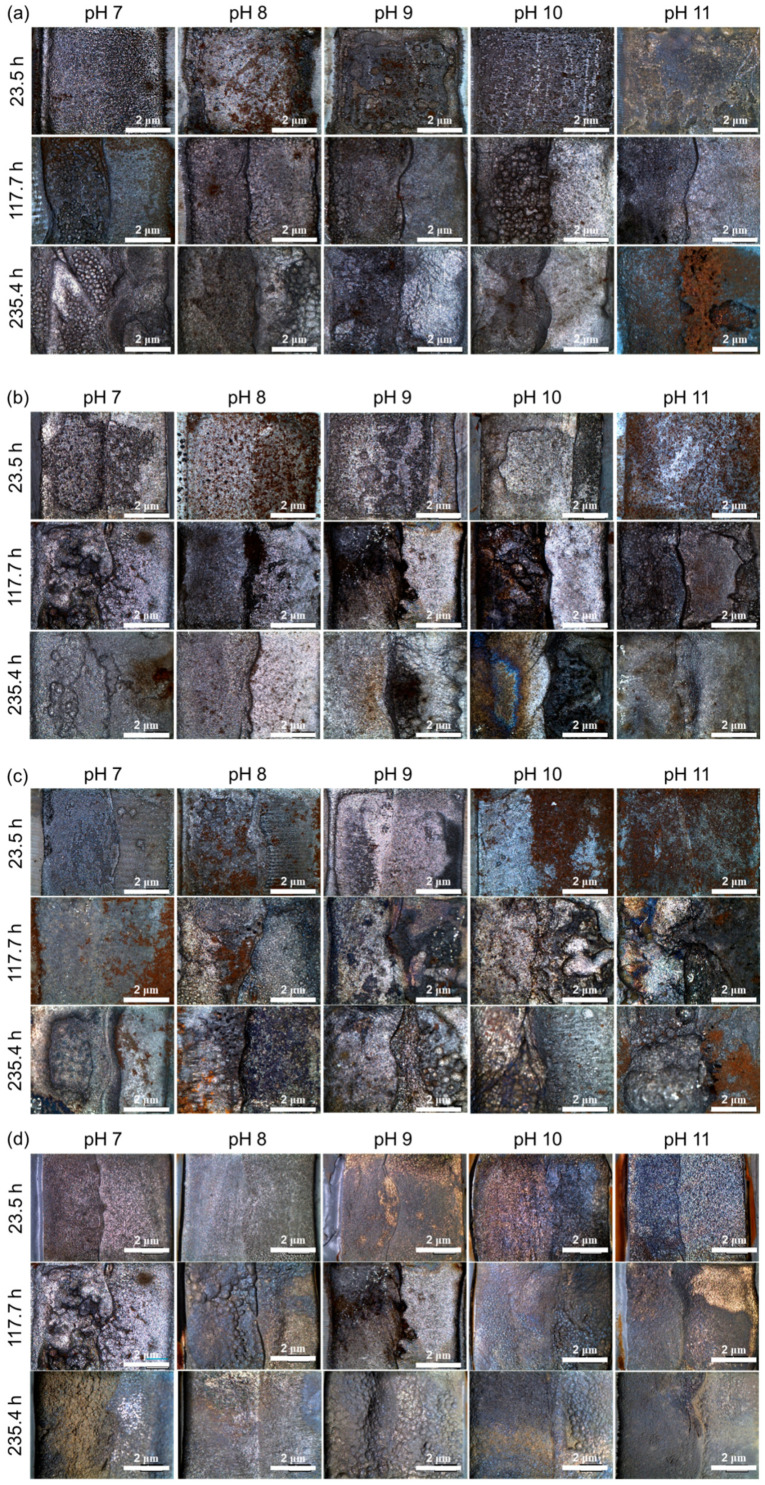
Photographs of weldment after the corrosion acceleration. DO: (**a**) 0, (**b**) 200, (**c**) 1000, and (**d**) 8000 ppb.

The testing conditions, including applied current density and test time, were determined based on the corrosion current density of the HAZ (4.304 μA/cm^2^) using Faraday’s law, and the results are summarized in Table 4. To accelerate the specimen’s corrosion, a current density of 4 mA/cm^2^ was applied. Figure 5 confirms that all specimens experienced uniform corrosion after the galvanostatic test. However, due to the more noble corrosion potential of the weld metal compared to the HAZ, a difference in the corrosion depth between the HAZ and weld metal was observed. Therefore, the corrosion depth of the HAZ was measured using a 3D profiler, and the results are presented in Table 5.

### 3.4. Regression Analysis and Validation of the Formula

Through multiple regression analysis using Minitab 19 software, the relationship between the experimental factors (pH, DO, and operating time) and corrosion depth was derived as follows.

Corrosion depth (μm) = −133 + 17.1 pH + 0.00072 DO + 125.2 Time − 7.95 pH × Time + 0.002921 DO × Time(4)

**Figure 6:**
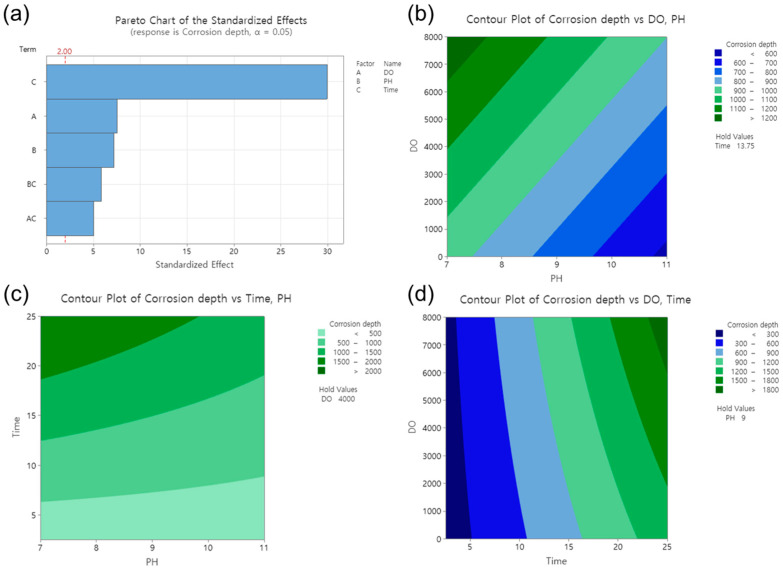
Effect of factors in response surface analysis. (**a**) Pareto chart of the standardized effects, contour plots of corrosion depth vs. (**b**) DO and pH, (**c**) operating time and pH, (**d**) DO and operating time.

The effect of various factors on corrosion depth was analyzed using RSM, and the results are presented in Figure 6. As shown in Figure 6a, the standardized effect of the factors appeared in the order of operating time, DO, and pH. The significance of each factor was determined using the Pareto chart, where the T-value was used to measure the coefficient’s significance with respect to the standard error [28,29,30,31,32]. The operating time was found to have the greatest influence on the corrosion depth. The bars that represent all factors on the Pareto chart cross the reference line at 2.00, indicating that these factors are statistically significant at the 0.05 level based on the current model [33].

To better understand the effect of each factor on the corrosion depth, two-dimensional (2D) surface contour plots were generated. Three different factors were examined, but the 2D plots could only represent two factors and the corrosion depth (response). Therefore, one factor had to be fixed as a constant value. Figure 6b presents the 2D plot demonstrating the effects of the DO and pH on the corrosion depth. With a fixed value of 13.75 years for time, the effect of the DO increased with the increasing DO concentration, resulting in a corrosion depth from 900 μm to 1200 μm. Conversely, the effect of pH on corrosion decreased with increasing the pH, resulting in a corrosion depth from 1000 μm to 600 μm. In Figure 6c, holding the DO constant at 4000 ppb, the corrosion depth increased from 500 μm to 2000 μm with an increase in the operating time. In Figure 6d, with a fixed pH value of 9, the corrosion depth increased from 300 μm to 1800 μm with an increase in the operating time. Consequently, in the DH water, the effect of the operating time had a greater effect on the corrosion depth than the pH and DO, especially within the pH range from 7 to 11 and the DO range from 0 to 8000 ppb.

The suitability of the formula was assessed through the coefficient of determination (R^2^) and analysis of variance (ANOVA). The R^2^ value explains the percentage of variation in the response variable [34,35]. R^2^ can have a value between 0 and 1, and a value close to 1 indicates a good relationship between experimental and predicted values [36]. In this formula, the R^2^ value was 0.95. ANOVA was used to study whether certain parameters had a substantial impact. To conduct ANOVA, the sum of squares is usually converted into contributions from the regression model and the residual error. In Table 6, the model, *p*-value, F-value, and associated probability values are reported to verify the significance of the model. The decision value is the *p*-value, which is usually set to <0.05 [37,38,39,40] to indicate model validation, and the F-value shows the impact of the process parameter on the response [41]. Therefore, when the *p*-value output from the ANOVA of this research is 0.00 (<0.05), there is a substantial difference between the variables. Here, all parameters have a substantial impact on the corrosion depth when their *p*-value is <0.05. Additionally, a lack-of-fit test was performed using ANOVA [42]. The lack-of-fit means that the multiregression model obtained through the experiments is not suitable to explain the data. In a lack-of-fit test, the null hypothesis is that “the model is good at explaining the data”. Thus, the regression model can be assessed to be meaningful only when the null hypothesis is not rejected because the *p*-value computed through the lack-of-fit test is greater than the significance level. As a result of the analysis, the R^2^ value was 0.95, and ANOVA verified that the *p*-value for the model and each coefficient showed a high confidence level at the significance level of 0.05. Therefore, the analysis and observation reiterate a good correlation between the experimental findings and the values predicted using the statistical model, which demonstrates the success of this model.

### 3.5. Corrosion Depth Prediction

**Figure 7:**
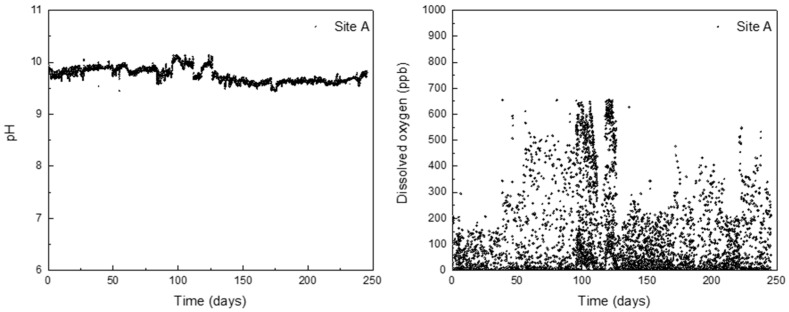
Actual monitoring data of pH and DO contained in DH water for 250 days.

Using the derived formula, the corrosion depth of the HAZ can be predicted based on the levels of corrosion factors. Since DH pipelines are buried in soil, it can be difficult to directly manage their corrosion. Therefore, the corrosion factors in DH water were monitored to indirectly predict the corrosion depth of the HAZ. The pH and DO levels in the DH water were measured every 30 min over a period of 250 days under operating conditions, and the results are presented in Figure 7. The pH was observed to remain stable between 9 and 10, which is within the management standard for DH water. However, the DO concentration exceeded the management standards of 200 ppb several times. Based on these results, the average values of corrosion factors were applied to the formula, and Table 7 displays the predicted depth of corrosion in the DH pipeline HAZ over time. This method is expected to provide an indirect means of examining the corrosion depth of the DH pipeline steel weldment.

## 4. Conclusions

In this research, galvanostatic acceleration was utilized to simulate the long-term corrosion of the DH pipeline weldment based on the corrosion factors. Following the measurement of corrosion depth, a multiple regression analysis was conducted to establish the relationship between corrosion depth and corrosion factors, namely, pH, DO, and operating time. The results of the R^2^ and ANOVA of the derived formula showed a high level of suitability. Additionally, an example of how the formula can be applied was presented. This approach can be extended to evaluate the corrosion of structures that are difficult to manage directly by measuring corrosion factors in the operating environment.

## Figures and Tables

**Table 1:** Composition of the DH pipeline materials (SPW400).

Composition (wt.%)				
C	Mn	P	S	Fe
≤0.25	1.00	≤0.04	≤0.04	Balance

**Table 2:** Composition of DH water.

pH	Temp.	NaCl (mg/L)	Mg(OH)_2_ (mg/L)	CaCO_3_ (mg/L)	NH_4_OH (mg/L)
10.0	60 °C	15.01	0.48	2.65	10.28

**Table 3:** Experimental conditions and calculated variables.

	β_a_ (V/dec.)	β_c_ (V/dec.)	E_corr_ (V_SCE_)	i_corr_ (A/cm^2^)
Heat-affected zone	0.084	0.039	−0.794	4.304 × 10^−6^
Weld metal	0.082	0.041	−0.807	2.569 × 10^−6^

**Table 4:** Computed testing conditions for the corrosion acceleration test.

In Service		Laboratory	
		Impressed Anodic Current Density	4.0 mA/cm^2^
Operating time	21,900 h (2.5 years)	Accelerated test time	23.5 h
	109,500 h (12.5 years)		117.7 h
	219,000 h (25 years)		235.4 h

**Table 5:** The corrosion depth of the HAZ measured using a 3D profiler.

DO (ppb)	Operating Time (Years)	pH	Corrosion Depth (μm)	DO (ppb)	Operating Time (Years)	pH	Corrosion Depth (μm)
0	2.5	7	126 ± 9	200	2.5	7	149 ± 10
		8	101 ± 12			8	126 ± 13
		9	121 ± 15			9	106 ± 14
		10	109 ± 14			10	108 ± 16
		11	169 ± 18			11	102 ± 13
	12.5	7	879 ± 32		12.5	7	936 ± 41
		8	598 ± 41			8	728 ± 35
		9	606 ± 45			9	732 ± 27
		10	694 ± 36			10	741 ± 34
		11	671 ± 28			11	671 ± 21
	25	7	1644 ± 86		25	7	1860 ± 101
		8	1079 ± 57			8	1279 ± 77
		9	1144 ± 62			9	1248 ± 68
		10	1074 ± 52			10	1200 ± 70
		11	1087 ± 43			11	1235 ± 60
1000	2.5	7	171 ± 14	8000	2.5	7	265 ± 15
		8	199 ± 21			8	204 ± 21
		9	146 ± 17			9	216 ± 13
		10	188 ± 26			10	230 ± 26
		11	121 ± 13			11	190 ± 9
	12.5	7	1040 ± 68		12.5	7	1240 ± 47
		8	845 ± 46			8	1019 ± 62
		9	767 ± 49			9	958 ± 42
		10	728 ± 38			10	962 ± 40
		11	679 ± 32			11	688 ± 21
	25	7	2248 ± 122		25	7	2604 ± 138
		8	1643 ± 88			8	2039 ± 121
		9	1391 ± 95			9	1892 ± 96
		10	1215 ± 68			10	1730 ± 102
		11	1289 ± 69			11	1422 ± 88

**Table 6:** Analysis of variance findings for the derived corrosion depth formula.

	Degree of Freedom	Sum of Square	Mean Square	F-Value	*p*-Value
Model	5	22,691,827	4,538,365	239.52	0.00
Linear	3	21,527,065	7,175,688	378.72	0.00
pH	1	1,185,230	1,185,230	62.55	0.00
DO	1	1,087,527	1,087,527	57.40	0.00
Time	1	19,056,253	19,056,253	1005.74	0.00
Interaction	2	1,122,202	561,101	29.61	0.00
pH × Time	1	804,149	804,149	42.44	0.00
DO × Time	1	449,929	449,929	23.75	0.00
Error	57	1,080,003	18,947	-	-
Lack of fit	54	1,066,591	19,752	4.42	0.122
Pure error	3	13,412	4471	-	-
Total	62	23,771,830	-	-	-

**Table 7:** Monitoring data and predicted corrosion depth.

	Averaged pH	Averaged DO (ppb)	Operating Time (Year)	Predicted Corrosion Depth (μm)
Site A	9.76	54.37	2.5	153.4
			12.5	631.0
			25.0	1228.1

## Data Availability

Data is contained within the article material.
